# Income-Related Mortality Inequalities and Its Social Factors among Middle-Aged and Older Adults at the District Level in Aging Seoul: An Ecological Study Using Administrative Big Data

**DOI:** 10.3390/ijerph19010383

**Published:** 2021-12-30

**Authors:** Minhye Kim, Suzin You, Jong-sung You, Seung-Yun Kim, Jong Heon Park

**Affiliations:** 1 Department of Sociology, College of Social Science, Changwon National University, Changwon-si 51140, Korea; minhyekim@changwon.ac.kr; 2 Inequality and Social Policy Institute, Gachon University, Seongnam-si 13120, Korea; youjs0721@gachon.ac.kr; 3 Department of Urban Society Research, Seoul Institute, Seoul 06756, Korea; francies@si.re.kr; 4 Big Data Steering Department, National Health Insurance Service, Wonju-si 26464, Korea; parkjh@nhis.or.kr

**Keywords:** administrative big data, districts in Seoul, ecological study, socioeconomic characteristics of small area, SII, RII, spatial analysis

## Abstract

This study investigated income-related health inequality at sub-national level, focusing on mortality inequality among middle-aged and older adults (MOAs). Specifically, we examined income-related mortality inequality and its social factors among MOAs across 25 districts in Seoul using administrative big data from the National Health Insurance Service (NHIS). We obtained access to the NHIS’s full-population micro-data on both incomes and demographic variables for the entire residents of Seoul. Slope Index of Inequality (SII) and Relative Index of Inequality (RII) were calculated. The effects of social attributes of districts on SIIs and RIIs were examined through ordinary least squares and spatial regressions. There were clear income-related mortality gradients. Cross-district variance of mortality rates was greater among the lowest income group. SIIs were smaller in wealthier districts. Weak spatial correlation was found in SIIs among men. Lower RIIs were linked to lower Gini coefficients of income for both genders. SIIs (men) were associated with higher proportions of special occupational pensioners and working population. Lower SIIs and RIIs (women) were associated with higher proportions of female household heads. The results suggest that increasing economic activities, targeting households with female heads, reforming public pensions, and reducing income inequality among MOAs can be good policy directions.

## 1. Introduction

Use of administrative income data in social science and social epidemiology has been limited [1]. However, population-based information on income from tax authorities has begun to be available for research starting from Scandinavian countries [2]. This provides an opportunity to make a breakthrough, as the improvement of the quality of individual income status and area-level factors such as Gini coefficient of income has been an important remaining task in health inequality research [3].

Meanwhile, reduction of health inequality is one of the main policy goals in the Health Plan, the biggest health promotion initiative in South Korea (hereafter Korea) [4]. However, insufficient attention has been paid to the health inequality among older adults despite the country’s rapid aging. This study focuses on the current and future elderly—middle-aged and older adults (MOAs) who were aged 45 years or more—across small areas (districts, *gu* in Korean) in Seoul. *Gu* works as the primary source of community identity and a basic unit of local public policy [3,5]. This study is a population-based study where an administrative big data from tax records are fully utilized to measure income-related mortality inequalities, as well as its variations and covariates across sub-national districts.

### 1.1. Population Aging and Health Inequality among MOAs

Seoul is an aging city with an aging rate of 16% in 2021 and is projected to reach an “aged” community in 2026 with more than 20% of its population consisting of older adults, surpassing the Organization for Economic Cooperation and Development (OECD) average of 19% [6]. The speed of aging is particularly high in Korea. It took only 17 years to become an aged society (14% of aging population) from an aging society (7% of aging population), whereas the same change took as long as 114 years for Western countries such as France [7]. In this context, life course sensitive approaches are becoming important. Moreover, MOAs have influenced by COVID-19 pandemic more strongly than younger generations [8]. However, few studies investigated health inequalities among MOAs [9]. Considering the full-fledged effect of socioeconomic status on health begin to take place from midlife, and epidemiological studies set the 45-year-old as a starting point of the midlife [10,11,12], this study investigated Seoul residents whose age were 45 or plus.

### 1.2. Health Inequality Research Using Population-Based Administrative Income Data

A great majority of existing studies on social determinants of health inequality rely on survey data regarding socioeconomic characteristics of individuals and areas, linking them to administrative mortality records [13,14,15,16]. In addition, the focus of socio-spatial analyses has been moved from national characteristics to sub-national areas as geographically smaller units are better fit to the notion of neighborhood [17]. 

However, most survey data do not contain sufficient cases to conduct an analysis by sub-national area, and simulation method using survey data provides limited information, making it necessary to use population-based administrative data [18]. In addition, survey data have limitations such as the inaccuracy of reported income and under-representation of the upper-class [2]. Values of Gini coefficient of income and poverty rate acquired from survey data were underestimated compared to those adjusted by administrative data [2]. 

Very few studies have used administrative income data to compute exact income groups of individuals or calculate area-level characteristics such as Gini coefficient of income [8,19,20]. A leading study by Chetty et al. [19] utilized administrative tax records in the United States (U.S.) to examine income-related differentials in life expectancy, variations across commuting zone (sub-national region), and its regional-level covariates. They reported that there were clear income-related gradients of life expectancy in the U.S. For instance, the 40-year-old men in the bottom income percentile had similar life expectancy for the same age men in Sudan, while the top 1 percent had higher life expectancy for men in all countries at the same age. In addition, low-income individuals showed higher area-level variations of life expectancy. However, this study did not provide comprehensive indicators of health inequality.

Filling this gap, another leading work by Decoster et al. [8] used tax data in Belgium to examine mortality inequality indices, i.e., Slope Index of Inequality (SII) and Relative Index of Inequality (RII) before and after COVID-19. Income gradient in mortality in terms of SII was substantially greater after the COVID-19 outbreak. When a multilevel regression approach was used, it was found that lower area-level income was significantly related to higher average mortality, controlling for individual income. However, this study did not conduct regression analysis regarding mortality gap.

Although area was not the focus, Kinge et al. [20] investigated income-related inequalities in avoidable mortality in Norway using the Norwegian Income Register and the Cause of Death Registry. They showed that the income-based gradient in avoidable mortality has constantly existed over time. However, health inequality was not related to income inequality index (Gini coefficient of income), hypothetically due to social changes such as pro-poor tax reform.

In the Korean context, previous studies used national health insurance contribution as a proxy for income because it was the only available income-related variable in the National Health Insurance Service (NHIS) database [5,16,21]. However, health insurance contributions are not an accurate marker of income for the residence-based insurance holders (or the self-employed insured) as opposed to workplace-based insurance holders (or employee-insured) [22]. The health insurance contribution among the workplace-based insurance holders are based on employee income, wages, and salaries. On the contrary, the health insurance contribution among the residence-based insurance holders are derived from information on income and wealth. Consequently, health insurance contribution for the residence-based insurance holders is greater than that for the workplace-based insurance holders with the same amount of income. In addition, business income is less transparent than earned income. Taken together, the health insurance contribution is less accurate proxy for individual income. 

This study used administrative big data from the NHIS of Korea, whose income data are transferred from the National Tax Service (NTS) [2]. This is the only database that contains information on income of the entire population at the individual level in Korea. This is the first Korean study to calculate individual income groups using a comprehensive information on wage, business income, interest, dividend, special occupational pension, national pension, and other income from administrative big data and relate them to health inequalities. In so doing, we examined spatial correlation using Moran’s I statistics as existing studies reported that health inequalities in districts could be spatially correlated [23].

### 1.3. Health Inequalities and District-Level Social Factors

For district-level local governments to implement appropriate health promotion policies, it is important to identify significant district-level social factors. Previous studies have used demographic and socioeconomic characteristics of districts [3,8,15,19,20,23,24,25,26]. Demographic factors include variables such as proportions of older adults, the divorced and unemployed population, and single-member households. Socioeconomic factors encompass (1) social inequality indicators such as the Gini coefficient of income, (2) class composition such as proportions of those with low education, (3) housing-related factors such as the proportion of renters, and (4) policy-related factors such as district-level health policy. These characteristics were entered into regression models independently or as composite indices (e.g., material deprivation index or area deprivation index).

The proportion of older adults was positively correlated with age-standardized mortality rate [15] and income-related inequality in quality-adjusted life expectancy [16]. Income inequality (measured as Gini coefficient) at the district level was significant in some studies—it was related to greater educational inequality of self-rated health [14] and inter-quintile difference in life expectancy [16]. Other studies showed that Gini coefficient of income was not significant in terms of avoidable mortality inequality by individual income [20]. Yet another study reported that Gini coefficient was important in life expectancy among higher income group, while not affecting life expectancy among lower income group [19].

The percentage of female household heads was a significant contributor to age-standardized mortality ratio across sub-districts [25] and income inequality in life expectancy across districts [16]. The proportion of single-member households, the appraised value of land/home, the percentage of social assistance recipients, the percentage of the low-educated, the percentage of immigrants, and the expenditure of local government had impact on health differentials [16,19,25]. Unemployment rates was significant in some studies [16], while the same effect was not found in other studies [19]. Policy initiatives such as Healthy City policy were effective in decreasing the prevalence of key diseases among districts [26].

Considering data availability, this study included calculable variables among the aforementioned demographic and socioeconomic factors in regression analyses. Spatial regression was conducted in the case where the spatial correlation was significant, in addition to ordinary least squares (OLS) regression.

### 1.4. Mortality Inequality and Measurements

Reducing mortality and gaps in mortality are the ultimate goals of epidemiological research and public health policies [27,28]. Mortality is the most frequently used measure of health in comparing different geographic areas and subgroups in a society [27,28]. In this study, the most recently available five years (2014–2018) of data were pooled to obtain stable mortality rate in districts [21,23,25]. 

The dependent variables were SII and RII of mortality, which are two of the most well-known rank-based indices of health inequality [29]. SII represents the absolute difference in health outcomes between the most advantaged and the most disadvantaged groups. It is the slope obtained by modeling the health outcome to be in a linear relationship with socio-economic status. RII represents the relative difference in health outcomes between the most advantaged and disadvantaged groups, by modeling the outcome to be log-linear by group status [29,30].

### 1.5. Aim of the Present Study

The aims of this study were to examine the (1) district-level differences of mortality inequality by individual income group and (2) social factors of district-level mortality inequality in Seoul using population-based administrative income data from NHIS and NTS.

## 2. Materials and Methods

### 2.1. Data

The data came from the NHIS income database and mortality data. The information in NHIS income database is transferred from NTS’ aggregate income tax, year-end settlement, and interim retirement settlement data of the employee-insured and public pensions (e.g., national pension and special occupational pension) data. NHIS and NTS data are administrative big data that cover the entire population in Korea (Seoul, in the case of the present study). These data are obtained from the process of administrative operations for various purposes such as registration and record-keeping [1].

The NHIS database provides other basic variables such as age, gender, residence, and information on death along with income data. Seoul residents aged 45 and above in the year 2014 were selected (men = 2,017,879; women = 2,239,916; sum = 4,257,795). To secure a sufficient number of deaths, we combined five years of data from 2014 to 2018 for all the individuals who resided in Seoul in 2014.

### 2.2. Study Design 

**Figure 1:**
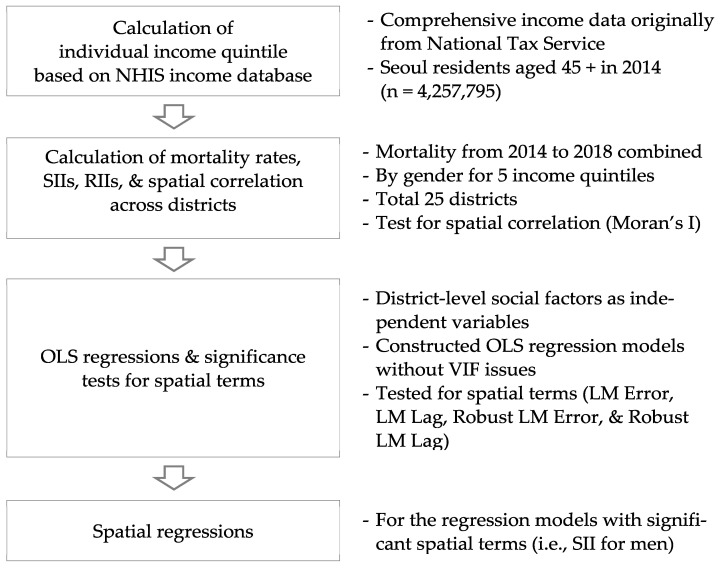
Study design.

This study first calculated individual income quintiles on the basis of the comprehensive income information from NHIS and NTS (Figure 1). Then, mortality rates by income quintiles and mortality indices (SIIs and RIIs) were computed for MOA men and women across 25 districts. Spatial correlations of SII and RII for men and women were tested using Moran’s I statistics.

After that, mortality indices (SIIs and RIIs) were regressed on social factors using districts as the unit of analysis. OLS regression models were firstly constructed with variables without variation inflation factor (VIF) issues, followed by significance tests for spatial terms (e.g., Lagrange multiplier (LM) error). If one of the spatial terms turn significant, spatial regression was additionally performed.

### 2.3. Income Groups

Income groups were computed on the basis of quintiles of real yearly income, averaged across 2014 to 2018 [29,30,31]. Income was the sum of wage and business income, as well as interest, dividend, special occupational pension, national pension, and other income. Real income is the income adjusted by the consumer price index. The reference year was 2015. Considering a large proportion of MOAs do not have market income due to retirement and gender division of labor, an equivalized household income was used instead.

### 2.4. SII and RII of Mortality

To compute SIIs and RIIs, we used ridit scores for income quintiles as the socioeconomic status variable. The income quintiles were based on real mean income for the five-year period of 2014 to 2018, where quintiles were computed for each gu, sex, and age group combination. These quintiles were converted into ridit scores such that if *p_i_* stands for the fraction of the population belonging to *i*th quintile, the ridit score for an individual belonging to *j*th quintile is 0.5*p_j_* + Σ*_i > j_ p_i_*. Thus, the ridit score represents individuals’ location in income distribution in terms of percentile from the bottom, assuming that each individual is precisely in the middle of the distribution within the quintile s/he belongs to. 

SII and RII were obtained as the function of the ridit score of income quintile, age, and age2. The equations are described in Equations (1) and (2), where the coefficient *α*_1_ in Equation (1) is the SII, and *β*_1_ in Equation (2) is the RII.

mortality = *α*_0_ + *α*_1_ ridit + *α*_2_ age + *α*_2_ age^2^ (1)

mortality = exp(*β*_0_ + *β*_1_ ridit + *β*_2_ age + *β*_2_ age^2^) (2)

Estimation of SIIs and RIIs was performed using SAS version 9.4′s PROC GEN- MOD procedure (SAS, Cary, NC, USA), following the code from Spiegelman et al. [31].

### 2.5. Demographic and Socioeconomic Characteristics of District

The independent variables were social factors, which were composed of demographic and socioeconomic factors. Considering previous studies [15,23,24,25] and the availability in the NHIS income database, we used the following district-level attributes from 2014 as demographic variables: the proportion of the population aged 65 or above among all residents (P65), the proportion of households with single older adult household head among all households (OSH), the proportion of women among heads of household aged 45 or above (FHH). Socioeconomic variables were as follows: poverty rate (PR), Gini coefficient of income (GC), the proportion of working population among those aged 45 or above (WP), and the proportion of pensioners among those aged 62 or above for National Pension (NP) and Special Occupational Pension (SOP). Poverty rate was calculated as the percentage of individuals whose equivalized household income was below 50 per cent of the median equivalized household income. Working population represents those who had either employee income (wages and salaries) or business income (income from self-employment).

**Figure 2:**
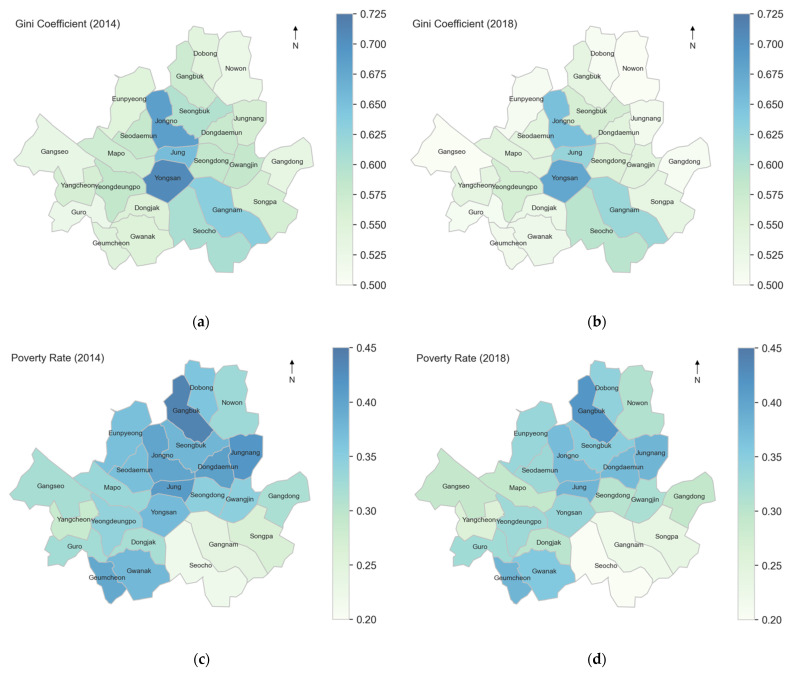
Gini coefficients and poverty rates in 2014 and 2018 across 25 districts in Seoul: (**a**) Gini coefficient of equivalized income in 2014; (**b**) Gini coefficient of equivalized income in 2018; (**c**) poverty rate in 2014; (**d**) poverty rate in 2018.

The average aging rate among 25 districts was 12.3% (Table 1). The average percent- age of old-age household heads among all households was 6.6%. Among all households with heads aged 45 or above, 30.3% were female on average. The average poverty rate and Gini coefficient were 34.7% and 0.579, respectively (Figure 2). The mean value of the proportion of the working population among MOA was 47.7%. The proportion of the population who received the NP was 31.6%, whereas that for the special occupational pension was as low as 4.1% on average.

### 2.6. Analytic Strategy 

First, SII and RII for men and women were regressed on socioeconomic and demographic factors using OLS. Different sets of independent variables were used, depending on the outcome variable. FHH was included only in regressions for women’s SII and RII. For income-related characteristics, absolute inequality (SII) was regressed on an absolute measurement (poverty rate), whereas relative inequality (RII) was regressed on a relative measurement (Gini coefficient) according to previous studies [15,23,24,25]. In the initial runs of OLS, all relevant variables were included (e.g., P65, OSH, WP, PR, SOP, and NP for SII among men) in the regression models. To avoid multicollinearity, the regression was run a second time after removing highly correlated variables with VIF greater than 10.

To test the need for spatial regression, we performed LM and robust Lagrange multiplier (robust LM) tests against the OLS models [32,33]. Two types of spatial models were considered. The first is the spatial error model (SEM), which is a linear model with a disturbance term that is a spatial autoregression on other districts’ disturbances. If *y* is the outcome variable, *X* is the matrix of independent variables, *β* is the vector of coefficients for *X*, and *ε* is the disturbance term, then the SEM is defined as

*y* = *Xβ* + *ε*, where *ε* = *λWε* + *μ*, (3)

that is, the disturbance of the regression model has a spatial structure. Here, *W* is the spatial weight matrix, *λ* is the coefficient for the spatial autoregression for the disturbance *ε*, and *μ* is the disturbance term for the spatial autoregression. This model is plausible if there is some spatial information that the set of independent variables do not account for.

The second spatial regression model considered is the spatial lag model (SLM), which is a mix of linear regression with spatial autoregression:

*y* = *ρWy* + *Xβ* + *ε* (4)

Here, *ρ* is the vector of coefficients for the spatial autoregression on the outcome variable *y*, and *ε* is the disturbance term for this mixed regressive-spatial autoregressive model. SLM is plausible if the outcome variable is spatially dependent on the outcome values of other regions. See [33] for more in-depth explanations of these models.

The spatial weighting method that produces the spatial weights matrix *W* can be considered a hyper-parameter. In this study, rook contiguity was used, so that all adjacent districts are given equal weights. This weighting method was deemed reasonable by the authors since each district is more likely to be related to adjacent districts, but not so much to nonadjacent districts, especially considering the fact that each district covers a large area. A more rigorous way of choosing the weighting method is left for future work.

If the LM and robust LM tests are significant with *p* < 0.05, the corresponding spatial model (either SEM or SLM) was fit. The final OLS and spatial models are presented in the Section 3. All analyses in this subsection were performed using PySAL [34]. 

## 3. Results 

### 3.1. Distribution and Spatial Correlation of SII and RII

Table 2 presents descriptive statistics of age-adjusted mortality rate per 10,000 people across districts, which were used as the basic information in calculating SII and RII. The mean value for the first (bottom) income quintile was 999, whereas that for the fifth (top) income quintile was as low as 382 for men. The least advantaged income group among men recorded highest mortality in Gangbuk-gu and the lowest mortality in Seocho-gu (Supplementary Material, Table S1). The difference between the highest and lowest mortality was 446 per 10,000 persons (Table 2), indicating considerable variation across districts.

In the case of women, the mean mortality rate for the first quintile was 416, while that for the fifth quintile was as small as 295. Similar to their male counterparts, women with the first income quintile showed the highest mortality in Gangbuk-gu and the lowest mortality in Seocho-gu (Supplementary Material, Table S2). The range was 167 per 10,000 persons (Table 2), which was smaller than men.

Nonetheless, these results suggested sizable regional differences of death rates across districts for both MOA men and women. Among the five income quintiles, the standard deviation of mortality by districts were highest for those with the first income quintile for men and women. For the detailed age-adjusted mortality rate by income quintile in each district, see the Supplementary Material (Tables S1 and S2).

SII per 10,000 persons ranged from 196 (Seocho-gu) to 492 (Gangbuk-gu) for men and 40 (Seocho-gu) to 155 (Jongno-gu) for women (Table 3). RII was smallest in Seongbuk- gu (3.10) for men and Guro-gu (1.12) for women, whereas it was the largest in Yongsan-gu (4.03) for men and Jongno-gu (1.62) for women.

**Figure 3:**
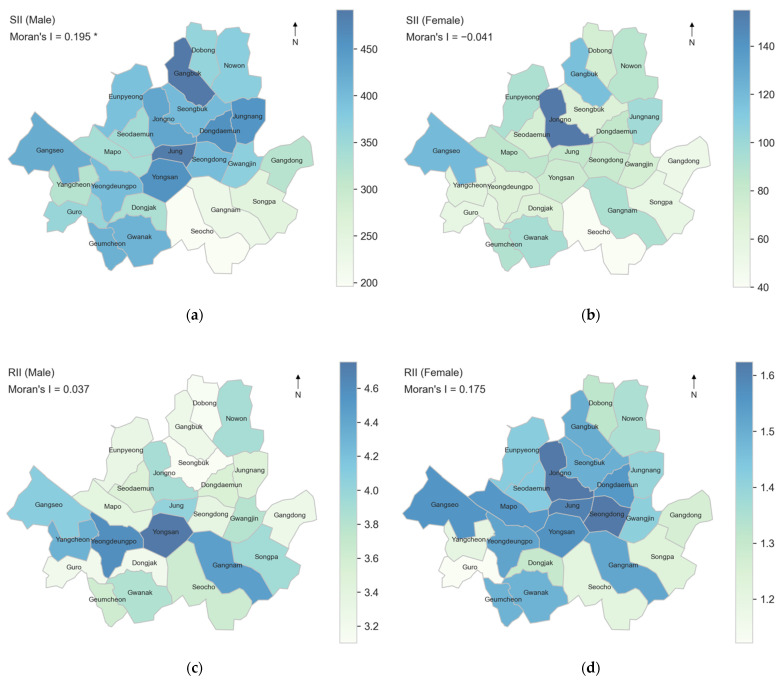
SIIs and RIIs by income quintile in 25 districts in Seoul for the period from 2014 to 2018 for men (left) and women (right) aged 45+. Weights used in computing Moran’s I are based on rook contiguity: (**a**) SII among men (per 10,000 people); (**b**) SII among women (per 10,000 people); (**c**) RII among men; (**d**) RII among women. *** *p <* 0.001, ** *p <* 0.01, * *p <* 0.05.

The spatial correlation was significant for men’s SII only (Moran’s I = 0.195, *p* < 0.05, Figure 3). Lower SIIs were found in the prosperous pan-Gangnam area (Seocho-gu, Gangnam-gu, and Songpa-gu) for men and women. Also notable is the high RII in Yongsan-gu for men, along with the low RII in Guro-gu and Seocho-gu for women (Figure 3).

### 3.2. OLS and Spatial Regression

#### 3.2.1. Procedure of OLS and Spatial Regression

After checking VIF and removing highly correlated items (Table 4), we entered selected variables into the final regression models (Table 5). We first entered all designated socio-economic factors in Table 4 in regression models for SII and RII for men and women. After checking VIF of each model, we removed highly correlated items one by one until the VIF issue was resolved. For example, in the case of SII for men, the population aged 65 or above among all residents (P65) was removed due to high correlation with the proportion of households with single older adult household head among all households (OSH). OSH had greater standardized coefficient than P65. Similarly, poverty rate (PR, smaller standardized coefficient than WP) was removed because of the high correlation with the proportion of working population among those aged 45 or above (WP, greater standardized coefficient than PR). The final model without VIF issue included OSH, WP, NP, and SOP as independent variables (Model 1, Table 5). Then, we fitted spatial regression model (Model 2) with the same set of independent variables as two (LM Lag, Robust LM Lag) of the spatial terms were marginally significant (Table 5). The same procedures were conducted for RII for men (Model 3, Table 5), SII for women (Model 4, Table 5), and RII for women (Model 5, Table 5).

#### 3.2.2. Results of OLS and Spatial Regression

The proportions of the working population and special occupational pensioners were significantly related to lower SII for men (Model 1, Table 5). A one percentage point increase of working population and special occupational pension earner corresponded to 8.7 and 16.9 decreases of SII per 10,000, respectively (*p* < 0.05). As the LM was significant, SLM was fitted (Model 2, Table 5). By accounting for spatial autocorrelation of the outcome, SII of men, we found that the effect of special occupational pension marginally increased (from -0.169 to −0.187), and that of the proportion of the working population remained almost the same (from −0.087 to −0.083), with improved statistical significance (*p <* 0.01).

RII for men was affected negatively by aging rate (1% point increase corresponds to 0.239 decrease of RII, *p* < 0.01) and positively by the Gini coefficient (0.01 increase to 0.076 increase of RII, *p* < 0.01) (Model 3, Table 5). Higher SII for women was associated with higher proportion of female household head (1% point increase to 9.1 increase of SII per 10,000, *p* < 0.05) (Model 4, Table 5). Greater RII for women was linked to higher proportion of female household head (1% point increase to 0.045 increase of RII, *p* < 0.05) and Gini coefficient (0.01 increase to 0.015 increase of RII, *p* < 0.05) (Model 5, Table 5). As for the RII for men, as well as SII and RII for women, no spatial model was fitted as the Moran’s I, LMs, and Robust LMs were not significant.

## 4. Discussion

### 4.1. Differential Mortality Rates, District-Level SII, and RII

#### 4.1.1. Summary of Findings

This study showed that income-related absolute and relative inequality in mortality were found across 25 districts among MOAs in aging Seoul. The lowest income group among men and women recorded greatest mortality in Gangbuk-gu and the lowest mortality in Seocho-gu. Among the five income groups, cross-district variance of mortality was greatest among the lowest income quintile with the range of 446 for men and 167 for women per 10,000. The SII ranged 196 (Seocho- gu) to 492 (Gangbuk-gu) per 10,000 for men and 40 (Seocho-gu) to 155 (Jongno-gu) per 10,000 for women. The RII varied from 3.10 (Seongbuk-gu) to 4.76 (Yongsan-gu) for men, and 1.12 (Guro-gu) to 1.62 (Jongno-gu) for women.

The mortality rates of the lowest quintile in Seocho-gu (744 for men and 320 for women per 10,000 persons) were close to those for the second quintile in Gangbuk-gu for men (693), and as low as for the highest quintile in Dobong-gu for women (320 per 10,000 persons, Supplementary Material Tables S1 and S2). This is because those with less and more income altogether had low mortality rates in Seocho-gu than other districts. The RII was lowest in Seongbuk-gu for men (3.10) and Guro-gu for women (3.22). As for Seongbuk-gu for men, this was because the mortality rates for the first and fifth quintiles were both high. In the case of Guro-gu for women, the fifth quintile had a relatively high mortality rate, whereas the first quintile showed a comparatively low death rate.

#### 4.1.2. Income-Related Mortality Gradient and Cross-District Differences

Overall, clear income gradient of mortality was found when administrative income data were used in deciding income groups, supporting the social gradient of health theory [35]. This is in line with the work of Chetty et al. [19] and Decoster et al. [8], which showed clear health gradient in terms of life expectancy in the U.S. and mortality SII and RII in Belgium.

The results showed that lower income group had greater district-level variations of mortality than higher income groups. This is similar to the work of Chetty et al. [19], wherein substantial area-level variation was found for low-income individuals, but a less obvious pattern was reported for high-income individuals. This suggests that the socioeconomic setting of small area and policies of local government have more impact on health among lower income groups.

Affluent districts such as Seocho-gu (one of the most representative districts in the famous Gangnam area) presented less mortality for the first income quintile and absolute health inequality (SII). In terms of the lower mortality among lower income group, the results echoed with Chetty et al. [19], where low-income individuals recorded higher life expectancy in wealthy communities such as California. In terms of mortality inequality, the results are similar to some Korean studies [16,21,36], whose results reported that prosperous communities showed a lower degree of inequality in healthy life expectancy for men and women. This suggests that wealthier community may provide more quality healthcare and effective intervention policies (e.g., smoking bans) [20].

#### 4.1.3. Spatial Correlation 

The spatial correlation was not strong—only the Moran’s I for men’s SII was marginally significant. A study on the spatial association of life expectancy among districts in Korea reported that spatial correlation (i.e., Moran’s I) was as large as 0.623 for men and 0.482 for women [23]. The finding of this study suggests that the spatial correlation of health inequality (i.e., SII and RII) can be less apparent than that of the average health status of a region. This result necessitates further studies on the meaning of spatial influence among adjacent communities, in terms of the difference between average health state and health differentials.

#### 4.1.4. Gender Differences

The mortality gradient was clearer among MOA men than women. Health inequality among men has been clearer in previous studies [16], as men’s social class is more straightforward, particularly among MOAs, in which large segments of women did not have individual income or occupational status [37].

#### 4.1.5. Significance of Using NHIS Income Data

Use of administrative income data in social science and social epidemiology has been rare compared to other types of data such as claims data, social network service data, and business data [29]. Previous Korean studies specifically used health insurance contributions as a proxy for actual income due to limitations of data availability, which produced a less accurate identification of income quintiles. Administrative big data of NHIS provided an opportunity to solve this problem by utilizing comprehensive information of income from NTS. The NHIS income database is becoming more accurate as the detection rate of one’s income by NTS has been improved [2]. The use of NHIS income database will help refine health inequality studies as well as in social science research [2].

### 4.2. Social Factors of District-Level SII and RII

#### 4.2.1. Gini Coefficient of Income

Relative inequalities of mortality for both genders were related to the Gini coefficient of income. This is in line with a previous study which showed that districts with high Gini coefficients in Seoul demonstrated greater relative health inequality by educational level [14]. Unlike the study of Lim et al. [16], which showed that the relationship between the Gini coefficient and inter-quintile health inequality was significant only for men, this study found that the relationship was similarly significant for both genders.

The effect of relative income inequality has been a controversial issue regarding the possible pathways linking relative income inequality to health inequality. Wilkinson [38] proposed income inequality hypothesis such that societies with greater income inequality had higher health inequality. However, some studies showed contrary results. For example, income inequality of area is not necessarily correlated with health inequality due to various reasons such as tax reform [20]. Other studies reported mixed results—in the work of Chetty et al. [19], Gini coefficient of income was related to higher income groups’ life expectancy but not related to lower income group’s health.

This study is the first attempt to calculate a more accurate income inequality index at the district level using administrative big data in Korea, showing that Gini coefficients are relevant indicators in Seoul. This suggests that local government may track Gini coefficients as part of health promotion policies.

#### 4.2.2. Pensions and Jobs

The results of this study suggest that providing a sufficient amount of pension benefits as can be seen in special occupational pension and providing works for MOA men would be a good policy direction to reduce absolute mortality inequality. Studies showed that older adults are divided by special occupational pensioners, national pensioners, and others in Seoul [2]. In 2018, the average total yearly income among special occupational pensioners was 41,226 U.S. dollars (USD) whereas for national pensioners it was around half that amount, at USD 20,365. Still, for those without either of these types of pension, the average yearly income was as low as USD 5608 [2]. 

Despite the purpose of public pension, the two public pension schemes in Korea work towards the direction of widening inequality. Historically, Korea’s public pension has been adopted for those who serve the developmental state first, such as civil servants and soldiers. The results of this study suggest that it is necessitated to increase the replacement rates of national pension or to expand basic old-age pension for the reduction of an absolute level of health inequality among MOA men. 

It is well known that the labor participation rate of Korean elderly is the highest among the OECD countries [39]. It seems that this has an impact on greater daily earnings of MOAs, particularly among the lower social class, which in turn, is linked to less mortality inequality. The results suggest that it is recommended to create jobs for MOAs through effective employment policies.

Meanwhile, pensions and employment rate were not significant for women. MOA women in Korea historically had less opportunity to enter formal labor force. This made national pension, not to mention special occupational pension, rather irrelevant for them. Previous studies showed that the effects of community characteristics on health inequality can differ between men and women. For example, the correlation between district-level characteristics and health inequality was stronger for men than for women in the study by Lim et al. [23].

#### 4.2.3. Female Household Head

The results suggest that intervening female household heads would be effective for reducing both absolute and relative health inequality among MOA women. This is because for women being a breadwinner of a family in Korea increases the likelihood of poverty. Among Seoul’s citizens, the income of the female household heads is only 51.5% of that of male household heads [2]. The proportion of female household head was also significant in predicting community-level health difference in other studies [25]. 

#### 4.2.4. Aging Rate

For MOA men, aging rate was significant in terms of relative health inequality, while this was not the case for MOA women. Health conditions of the lowest income quintile among male older adults are particularly poor, although those of low-income female older adults are not necessarily so [40]. Increases in the proportion of those who reach older ages were a significant contributor to health inequality among MOA men. Hence, it can be effective for local government to keep track of and target male older adults.

### 4.3. Limitations 

Even though NHIS income database is the only source of data containing income of the entire Korean population among all administrative data [2], it had some limitations such as unavailability of financial income below the 20 million Korean won mark and daily worker’s income. These information will begin to be included in NHIS database for the reform of the insurance contribution system starting 2022 [2]. 

Korean publications recommended 30 or more cases for sub-national studies [3]. This study included 25 cases, as this was the official number of administrative districts in Seoul, and NHIS does not allow the publication of studies using smaller administrative units (e.g., dongs). However, conducting analysis using smaller areas can be beneficial, as this better fits the notion of community. Studies on smaller areas allows more cases and a larger variation, which allows for a more reliable analysis.

In addition, it was not possible to disentangle contextual and compositional effects as this study was an exploratory ecological study. Further research is necessary to differentiate the effects of community-level demographic and socioeconomic characteristics from those at an individual level.

As mentioned in the methodology section, the choice of weighting method for generating the spatial weights matrix can be improved. Various weighting methods such as distance-based weighting or K-nearest neighbors weighting methods can be considered and compared via cross-validation.

It will be useful to include more independent variables. On average, 4.64 area-level independent factors were used in previous studies [3]. More refined variables can be produced if the NHIS income database is linked to other key administrative datasets. For example, educational level is a significant feature of an area [25], and this can be analyzed if data from the Ministry of Education are linked. While data linkage across different agencies and ministries requires much time to coordinate and execute, it can be achieved on a longer timeline.

### 4.4. Policy Implications 

National health initiatives such as Health Plan 2030 in Korea mandates local governments to establish effective action plans [4]. However, aside from some programs addressing harmful habits such as smoking and drinking, the relevance of the programs mentioned in the action plans to social policies remain abstract [4]. Although the plan highlights rapid aging and life-course perspectives, policies targeting MOAs are unclear. The results of this study can inform district governments that monitoring and intervening in fundamental social dimensions may result in reduced health inequality, although the direction of causality must be further studied. Tracking social indices such as the Gini coefficient of income using administrative data can further enhance the effectiveness of health promotion policies. 

## 5. Conclusions

The first income quintile (least level of income) demonstrated greatest mortality and variation by districts in Seoul. More prosperous districts had lower mortality rates for the first income group and less level of mortality inequality in terms of SII. The spatial correlation was marginally significant for men’s SII only. Lower Gini coefficient of income was significantly related to lower RII for MOA men and women, suggesting the importance of examining Gini coefficient of income as parts of local health policies for sub-national districts. In addition, increasing labor force participation, reforming public pensions, and intervening female household heads can be effective policies for reducing mortality inequalities among MOAs.

## Figures and Tables

**Table 1:** Descriptive statistics for demographic and socio-economic factors across 25 districts in Seoul in 2014 for men and women.

Variables		Mean	SD	Min.	Max.	Max.−Min.
Demographic factors	P65 ^1^	0.123	0.016	0.099	0.151	0.052
	OSH ^2^	0.066	0.013	0.045	0.091	0.046
	FHH ^3^	0.305	0.019	0.268	0.336	0.068
Socio-economic factors	PR ^4^	0.347	0.054	0.220	0.438	0.218
	GC ^5^	0.579	0.047	0.525	0.707	0.183
	WP ^6^	0.477	0.039	0.413	0.569	0.156
	NP ^7^	0.316	0.018	0.282	0.345	0.063
	SOP ^8^	0.041	0.016	0.023	0.091	0.068

^1^ P65: proportion of population aged 65 or above; ^2^ OSH: proportion of households with single older adult household head; ^3^ FHH: proportion of women among heads of household aged 45 or above; ^4^ PR: poverty rate, defined as proportion of population below 50% of median equivalized income; ^5^ GC: Gini coefficient; ^6^ WP: proportion of working population among those aged 45 or above; ^7^ NP: proportion of national pension earners among those aged 62 or above; ^8^ SOP: proportion of special occupational pension earners among those aged 62 or above.

**Table 2:** Distribution of age-adjusted mortality rate per 10,000 persons across 25 districts in Seoul for the period of 2014 to 2018 for men and women aged 45+.

Income Quintile	Male			Female						
	Mean	SD	Min.	Max.	Max.−Min.	Mean	SD	Min.	Max.	Max.−Min.
First	999	118	744	1190	446	416	43	320	487	167
Second	597	48	481	693	212	319	22	269	350	81
Third	523	57	382	641	259	313	23	270	357	87
Fourth	449	54	312	532	221	308	22	247	346	99
Fifth	382	60	262	489	227	295	31	235	373	139

**Table 3:** SIIs per 10,000 and RIIs of 25 districts of Seoul for the period from 2014 to 2018 for men and women.

	Male	Female	Male	Female
Jongno-gu	430	155	3.90	1.62
Jung-gu	488	82	4.03	1.60
Yongsan-gu	457	77	4.76	1.57
Seongdong-gu	399	79	3.36	1.62
Gwangjin-gu	369	72	3.80	1.41
Dongdaemun-gu	459	83	3.52	1.55
Jungnang-gu	453	98	3.47	1.40
Seongbuk-gu	402	68	3.10	1.50
Gangbuk-gu	492	117	3.27	1.50
Dobong-gu	359	73	3.12	1.32
Nowon-gu	371	87	3.90	1.35
Eunpyeong-gu	392	92	3.31	1.42
Seodaemun-gu	344	72	3.50	1.43
Mapo-gu	339	85	3.38	1.55
Yangcheon-gu	321	62	4.32	1.20
Gangseo-gu	421	120	4.09	1.56
Guro-gu	357	57	3.22	1.12
Geumcheon-gu	415	90	3.61	1.49
Yeongdeungpo-gu	399	66	4.59	1.52
Dongjak-gu	331	66	3.21	1.30
Gwanak-gu	412	95	3.83	1.49
Seocho-gu	196	40	3.64	1.21
Gangnam-gu	225	92	4.46	1.52
Songpa-gu	253	56	3.92	1.23
Gangdong-gu	318	51	3.26	1.26

**Table 4:** Pearson correlation coefficients for SII, RII, demographic factors, and socio-economic factors across 25 districts in Seoul.

	SII		RII		P65	OSH	FHH	PR	GC	WP	SOP
	M	F	M	F							
P65 ^1^	0.72 **	0.46 *	−0.21	0.55 **	-	-	-	-	-	-	-
OSH ^2^	0.77 **	0.51 **	−0.05	0.54 **	0.93 **	-	-	-	-	-	-
FHH ^3^	0.73 **	0.61 **	0.04	0.72 **	0.79 **	0.76 **	-	-	-	-	-
PR ^4^	0.92 **	0.53 **	−0.24	0.54 **	0.81 **	0.80 **	0.73 **	-	-	-	-
GC ^5^	0.17	0.25	0.51 **	0.52 **	0.41 *	0.44 *	0.36	0.14	-	-	-
WP ^6^	−0.92 **	−0.44 *	0.32	−0.51 **	−0.80 **	−0.76 **	−0.71 **	−0.97 **	−0.03	-	-
SOP ^7^	−0.88 **	−0.39	0.2	−0.42 *	−0.54 **	−0.61 **	−0.57 **	−0.85 **	0.11	0.86 **	-
NP ^8^	−0.68 **	−0.44 *	−0.04	−0.58 **	−0.78 **	−0.90 **	−0.79 **	−0.68 **	−0.44 *	0.66 **	0.56 **

^1^ P65: Proportion of population aged 65 or above; ^2^ OSH: proportion of households with single older adult household head; ^3^ FHH: proportion of women among heads of household aged 45 or above; ^4^ PR: poverty rate, defined as proportion of population below 50% of median equivalized income; ^5^ GC: Gini coefficient; ^6^ WP: proportion of working population among those aged 45 or above; ^7^ SOP: proportion of special occupational pension earners among those aged 62 or above. ^8^ NP: proportion of national pension earners among those aged 62 or above; *** *p <* 0.001, ** *p <* 0.01, * *p <* 0.05.

**Table 5:** OLS and spatial regressions for SII and RII on demographic factors and socio-economic factors across 25 districts in Seoul for men and women.

Outcome	Male						Female			
	SII				RII		SII		RII	
Model	Model 1 (OLS)		Model 2 (SLM)		Model 3 (OLS)		Model 4 (OLS)		Model 5 (OLS)	
Variable	*β*	SE	*β*	SE	*β*	SE	*β*	SE	*β*	SE
Intercept	0.072	0.035	0.083 **	0.029	4.254	3.191	−0.058	0.037	−0.255	1.296
NP ^1^	0.016	0.069	0.006	0.056	−5.615	7.013	0.070	0.06	−0.476	2.647
SOP ^2^	−0.169 *	0.067	−0.187 **	0.055	−5.646	6.458	−0.038	0.053	−3.199	1.895
WP ^3^	−0.087 *	0.034	−0.083 **	0.027	-	-	0.023	0.028	-	-
OSH ^4^	0.139	0.114	0.175	0.093	-	-	0.105	0.089	−4.447	3.819
FHH ^5^	-	-	-	-	-	-	0.091 *	0.04	4.497 *	1.759
GC ^6^	-	-	-	-	7.612 **	2.013	-	-	1.535 *	0.562
P65 ^7^	-	-	-	-	−23.939 **	7.765	-	-	-	-
*Wy*	-	-	−0.311 *	0.146	-	-	-	-	-	-
R-squared	0.898	-	0.917	-	0.525	-	0.427	-	0.653	-
Adjusted R-squared	0.878	-	0.919 †	-	0.43	-	0.277	-	0.561	-
Moran’s I of residuals	−0.050	-	-	-	0.032	-	−0.101	-	−0.100	-
LM (error)	0.126	-	-	-	0.051	-	0.505	-	0.497	-
LM (lag)	4.132 *	-	-	-	0.147	-	1.129	-	0.143	-
Robust LM (error)	0.748	-	-	-	0.776	-	0.157	-	2.416	-
Robust LM (lag)	4.754 *	-	-	-	0.872	-	0.781	-	2.062	-

^1^ NP: Proportion of national pension earners among those aged 62 or above; ^2^ SOP: proportion of special occupational pension earners among those aged 62 or above; ^3^ WP: proportion of working population among those aged 45 or above; ^4^ OSH: proportion of households with single older adult household head; ^5^ FHH: proportion of women among heads of household aged 45 or above; ^6^ GC: Gini coefficient; ^7^ P65: proportion of population aged 65 or above; † Spatial pseudo R-squared. *** *p <* 0.001, ** *p <* 0.01, * *p <* 0.05.

## Data Availability

Data sharing not applicable as this is administrative data limited to qualified users.

## Supplementary Materials

The following supporting information can be downloaded at: https://www.mdpi.com/article/10.3390/ijerph19010383/s1, Table S1: Age-adjusted mortality rate per 100,000 people by income quintile in 25 districts in Seoul for the period of 2014 to 2018 for men aged 45+ (*n* = 2,017,879); Table S2: Age-adjusted mortality rate per 100,000 people by income quintile in 25 districts in Seoul for the period of 2014 to 2018 for women aged 45+ (*n* = 2,239,916).

## Abbreviations

FHH	Proportion of women among heads of household aged 45 or above
GC	Gini coefficient
MOAs	Middle-aged and older adults
NHIS	National Health Insurance Service
NP	Proportion of national pension earners among those aged 62 or above
NTS	National Tax Service
OECD	Organization for Economic Cooperation and Development
OLS	Ordinary Least Squares
OSH	Proportion of households with single older adult household head
P65	Proportion of population aged 65 or above
PR	Poverty rate, defined as proportion of population below 50% of median income, in equivalized
RII	Relative Index of Inequality
SEM	Spatial error model
SII	Slope Index of Inequality
SLM	Spatial lag model
SOP	Proportion of special occupational pension earners among those aged 62 or above
U.S.	United States
USD	U.S. dollar
VIF	Variation inflation factor
WP	Proportion of working population among those aged 45 or above
