# Supplementary material for: Income-Related Mortality Inequalities and Its Social Factors among Middle-Aged and Older Adults at the District Level in Aging Seoul: An Ecological Study Using Administrative Big Data
Source: Int J Environ Res Public Health. 2021 Dec 30;19(1):383. doi: 10.3390/ijerph19010383 (PMC8751095; doi:10.3390/ijerph19010383)
Supplement: Supplementary file 1 [file ijerph-19-00383-s001.zip › ijerph-1467655-supplementary.pdf]

Table S1. Age-adjusted mortality rate per 100,000 people by income quintile in 25 districts in Seoul for the period of 2014 to 2018 for men aged 45+ (n=2,017,879)

	1st quintile (Lowest)	2nd quintile	3rd quintile	4th quintile	5th quintile (Highest)
Seoul	9,922	5,948	5,223	4,390	3,617
Jongno-gu	10,928	6,380	5,531	4,602	3,543
Jung-gu	10,998	6,239	5,382	4,815	3,582
Yongsan-gu	11,380	5,729	5,393	3,996	3,108
Seongdong-gu	10,303	5,961	5,765	4,740	3,980
Gwangjin-gu	9,548	6,036	5,380	4,525	3,467
Dongdaemun-gu	11,325	6,222	5,578	5,302	4,374
Jungnang-gu	10,839	6,339	6,111	5,323	4,446
Seongbuk-gu	10,376	6,090	5,511	4,827	4,323
Gangbuk-gu	11,896	6,934	6,408	5,171	4,612
Dobong-gu	9,862	5,994	5,324	4,712	4,437
Nowon-gu	11,431	6,320	5,205	4,825	4,563
Eunpyeong-gu	10,236	5,945	5,411	4,855	3,954
Seodaemun-gu	10,388	5,914	4,961	4,293	3,621
Mapo-gu	9,846	6,128	5,328	4,331	3,620
Yangcheon-gu	9,606	6,145	4,912	4,386	3,840
Gangseo-gu	10,885	6,424	5,478	4,669	4,184
Guro-gu	8,811	5,950	5,413	4,727	3,689
Geumcheon-gu	10,314	6,273	5,099	4,730	4,891
Yeongdeungpo-gu	9,734	5,595	5,169	4,003	2,998
Dongjak-gu	8,823	5,877	4,849	4,177	3,545
Gwanak-gu	10,124	6,188	5,302	4,455	3,759
Seocho-gu	7,435	4,812	3,823	3,305	2,895
Gangnam-gu	7,795	4,961	3,994	3,117	2,622
Songpa-gu	7,860	4,980	4,177	3,823	3,068
Gangdong-gu	8,985	5,853	5,279	4,489	4,497

Table S2. Age-adjusted mortality rate per 100,000 people by income quintile in 25 districts in Seoul for the period of 2014 to 2018 for women aged 45+ (n=2,239,916)

	1st quintile (Lowest)	2nd quintile	3rd quintile	4th quintile	5th quintile (Highest)
Seoul	4,128	3,185	3,120	3,091	2,879
Jongno-gu	4,248	3,049	3,182	3,131	2,399
Jung-gu	4,604	3,257	2,987	2,903	2,720
Yongsan-gu	4,133	3,184	3,098	2,809	2,621
Seongdong-gu	4,340	3,455	2,935	3,160	3,013
Gwangjin-gu	3,871	3,083	3,107	3,314	2,800
Dongdaemun-gu	4,711	3,462	3,196	3,181	3,070
Jungnang-gu	4,440	3,343	3,572	3,459	3,048
Seongbuk-gu	4,363	3,248	3,098	3,228	2,875
Gangbuk-gu	4,874	3,495	3,501	3,287	3,020
Dobong-gu	4,135	3,310	3,251	3,185	3,197
Nowon-gu	4,556	3,323	3,355	3,129	3,280
Eunpyeong-gu	4,204	3,420	3,023	3,081	3,141
Seodaemun-gu	4,425	3,120	3,214	3,010	2,917
Mapo-gu	4,181	2,989	3,036	2,950	2,594
Yangcheon-gu	3,829	3,336	3,356	3,220	3,259
Gangseo-gu	4,674	3,291	3,116	3,272	3,108
Guro-gu	3,760	3,095	3,322	3,444	3,207
Geumcheon-gu	4,605	3,413	3,382	2,999	3,732
Yeongdeungpo-gu	3,990	2,907	2,699	2,937	2,828
Dongjak-gu	3,872	3,212	2,803	3,099	2,996
Gwanak-gu	4,181	3,207	3,079	3,023	3,095
Seocho-gu	3,202	2,813	2,867	2,761	2,485
Gangnam-gu	3,471	2,689	2,712	2,468	2,346
Songpa-gu	3,414	2,751	2,981	2,988	2,997
Gangdong-gu	3,800	3,277	3,353	3,053	2,939
